# “I Just Feel Like the Teacher Understood Me, and She Knew What I Needed”: School Experiences of Autistic Students from Diverse Backgrounds

**DOI:** 10.1177/23969415251377973

**Published:** 2025-10-15

**Authors:** Jodie Smith, Aspasia Stacey Rabba, Georgia Coverdale, Poulomee Datta, Gabrielle Hall, Melanie Heyworth, Kristelle Hudry, Wenn Lawson, Rozanna Lilley, Elizabeth Pellicano

**Affiliations:** 1 Macquarie School of Education, Macquarie University, Sydney, Australia; 2 School of Allied Health, Human Services and Sport, La Trobe University, Melbourne, Australia; 3 Department of Psychology, Counselling and Therapy, School of Psychology and Public Health, La Trobe University, Melbourne, Australia; 4 School of Educational Psychology and Counselling, Faculty of Education, Monash University, Melbourne, Australia; 5 School of Psychological Science, The University of Western Australia, Perth, Australia; 6 The Kids Research Institute Australia, The University of Western Australia, Perth, Australia; 7 Reframing Autism, Sydney, Australia; 8 Curtain Autism Research Group (CARG), Curtain University, Perth, Australia; 9 Department of Clinical, Educational and Health Psychology, University College London, London, United Kingdom

**Keywords:** Autistic students, educational experiences, cultural diversity, qualitative research, teachers

## Abstract

**Background and aims:**

Gathering Autistic young people's testimony is critical for understanding their lived experience of education and designing settings in which these students can thrive. Despite increasing knowledge in this field, we lack perspectives from a broad range of Autistic students which necessarily limits our ability to build inclusive, supportive environments for all. This study explored the educational experiences of preschool and school-aged Autistic students from diverse age groups, backgrounds, and educational settings.

**Methods:**

Thirty-six Autistic students (aged 4–18 years) from Chinese, Vietnamese, Somali, Lebanese, and White Australian backgrounds shared their thoughts and experiences of their education. Through semi-structured interviews, students told their stories using words and pictures. Interview transcripts were analyzed using reflexive thematic analysis.

**Results:**

Students described experiencing significant overwhelm within education settings, which led them to value access to safe spaces and having autonomy over decisions in their school day. A strong sense of fairness and justice was reported with students frustrated by inequitable application of school rules, as well as being discouraged by educators’ low expectations of them. Students preferred teachers who were clear and direct in their communication and genuinely cared about them as individuals. Students were mindful of others’ differences and perspectives, striving for mutual respect and friendship with their peers.

**Conclusions:**

Findings from this research indicate that to thrive academically, emotionally and socially, Autistic students need thoughtfully designed education settings with high expectations for every student, together with individualistic care from teachers.

**Implications:**

Our findings reinforce how classroom design and education practices must consider the needs of all students for Autistic students to thrive. From a practice perspective, promoting student autonomy around aspects of their educational environment—such as the ability to use headphones in class or provision of spaces in which to retreat to prevent or manage sensory/social overwhelm—could be “quick wins” for schools wanting to foster safer, more secure settings for Autistic learners. Broadly, educators should aim to embed as much certainty as possible into Autistic students’ educational environments to lay a solid foundation for learning. This foundation is likely to be most effective when educators are partners in discovery with each individual Autistic student, seeking to understand their unique strengths, needs, personalities and identities, and build trusting student–teacher relationships. While our research examined the perspectives of a diverse range of Autistic students, future research should attempt to elicit the educational experiences of both younger Autistic children (e.g., preschoolers) and non- or semi-speaking children, exploring methods suited to this purpose.

A growing body of research details Autistic students’ experiences of education from their own perspectives, including what they want and need from teachers and schools in order to learn effectively (Cohen et al., 2022; Hasson et al., 2022; Humphrey & Lewis, 2008; Lynam et al., 2024; Makin et al., 2017; Williams et al., 2019). Eliciting Autistic young people's testimony is critical for understanding their lived experience of education systems (Carrington & Graham, 2001; Davidson, 2010) and for being able to design and implement education settings in which Autistic students can thrive. Yet, despite increasing knowledge in this field, we lack perspectives from a broad range of Autistic students—across different age groups, gender identities, education placements and ethnic backgrounds (Lynam et al., 2024)—which necessarily limits our ability to build inclusive, supportive environments for all. The current study aimed to address this gap, by inviting a group of Autistic students from diverse backgrounds to use their words and drawings to share viewpoints often previously overlooked in existing studies on educational experiences.

The vast majority of past studies report a myriad of challenges faced by Autistic students at school across their educational careers (Brede et al., 2017). Such challenges include overwhelming sensory environments (Friedlander, 2010; Horgan et al., 2023; Howe & Stagg, 2016; Williams et al., 2019), managing increasing social demands (Hedges et al., 2014; Horgan et al., 2023; Makin et al., 2017), navigating transitions and unpredictability (Danker et al., 2016; Horgan et al., 2023; Humphrey & Lewis, 2008), and feeling isolated and different from peers (Humphrey & Lewis, 2008; Makin et al., 2017; Williams et al., 2019). Indeed, difficulties developing quality peer relationships have been frequently reported (Calder et al., 2013; Horgan et al., 2023; Humphrey & Lewis, 2008; Makin et al., 2017), with many Autistic students describing negative experiences with classmates (Humphrey & Lewis, 2008; Humphrey & Symes, 2010), including bullying, victimization and stigmatization (Cohen et al., 2022; O’Hagan et al., 2021; Williams et al., 2019). Autistic students have also described restricted and/or poor interactions with their teachers, in part driven by limited knowledge and (mis)understanding of autism (Cohen et al., 2022; Horgan et al., 2023; O’Hagan et al., 2021; White et al., 2023), which teachers themselves readily admit (Roberts & Webster, 2020; Robertson et al., 2003). These challenges likely accumulate, contributing to increasing Autistic students’ stress and anxiety, and reducing wellbeing (Horgan et al., 2023; O’Hagan et al., 2021) as well as poorer longer-term outcomes.

In the few instances in which Autistic students report positive school experiences, they have repeatedly highlighted the importance of strong, interpersonal connections with peers and school staff, and of environments that help them learn and feel included. Autistic young people state that they find it helpful when teachers treat them fairly, actively listen and get to know them as individuals (Gulec-Aslan et al., 2013; Horgan et al., 2023; White et al., 2023), as well as provide tailored supports, delivered respectfully (Hasson et al., 2022; Humphrey & Lewis, 2008; White et al., 2023). Firm, even-handed teachers who make learning fun and include students’ interests are also valued by Autistic students (White et al., 2023; Wood, 2019). Similarly, structured but flexible learning environments are important, including flexibility around assignment deadlines and workload (Carrington & Graham, 2001; Saggers et al., 2012), and allowing regular breaks (Hasson et al., 2022). Indeed, what works for autistic students is flexibility and individualization regardless of the education context—with these being specific benefits of homeschooling over traditional education institutions (Heyworth et al., 2021; O’Hagan et al., 2021).

When described, the demographic characteristics in existing reports (i.e., see Steinbrenner et al., 2022) on the education experiences of Autistic students have tended to be rather narrow in scope—that is, focused on older Autistic boys in mainstream settings, from predominantly (or exclusively) white, middle-income families (Brede et al., 2017; Hedges et al., 2014; Makin et al., 2017; Saggers, 2015). Lynam et al. (2024) conducted a recent scoping review of Autistic students’ experiences finding that, in 36 studies included, most participants were male, very few reported on primary school children's experiences and only one included children in special educational settings transitioning into mainstream schools. Further, children's ethnic/racial backgrounds were not reported in the review, an omission highlighting the limited research focus on the intersectional experiences of Autistic students.

This limited research is a clear gap in the literature, impeding understanding of how diverse characteristics, such as gender and ethnicity, shape the education experiences of Autistic students. Existing biases within educational institutions continue to include gender stereotyping (Doornkamp et al., 2022; McCoy et al., 2021) and discrimination based on a student's racial and ethnic background (Childs & Wooten, 2022; Yusuf et al., 2022)—including for Autistic populations (Golson et al., 2022; Taylor & Henninger, 2015). We must understand the broader Autistic student experience in order to build inclusive, supportive environments for everyone.

The current study sought to examine the educational experiences of a large and diverse group of Autistic students from varying racial/ethnic background**s**, genders, ages, and school settings—spanning mainstream and specialist provision, and kindergarten, primary and secondary schools. Specifically, we conducted semi-structured interviews to explore Autistic students’ perspectives about their school experiences in order to understand which factors may effectively support their participation and achievement. To mitigate the limitation of a singular data collection method (Lewis & Porter, 2004), we asked students to share their thoughts and experiences using both words and pictures.

## Method

### Participants

Autistic students were recruited from two Australian states (Victoria and New South Wales) as part of a broader study on home–school partnerships for underserved groups; Autistic students from minority-ethnic backgrounds (specifically from Chinese, Vietnamese, Somali, and Middle Eastern communities), and Autistic students whose parent(s) also identified as Autistic. Further information about the broader study is available (Rabba et al., 2024; Smith, Rabba, Ali et al., 2023; Smith, Rabba, Cong, et al., 2023; Smith, Rabba, Dang et al., 2023).

Recruitment directly targeted parents as participants, via formal and informal networks of the study team, with all study information being available in English and home languages relevant to the aforementioned minority-ethnic groups. Supportive interpreters were also available if needed**.** Parents were invited to the study if they had children with a clinical autism diagnosis who were currently engaged in formal education (i.e., kindergarten, primary or secondary schooling). During the consenting process, participating parents were asked whether their Autistic children would also like to be interviewed to share their own education experiences. Those children were then subsequently invited and asked to provide verbal or written consent to participate. All participating students were reimbursed with a AUD$25 voucher.

Thirty-six Autistic students aged 4–18 years (*M* = 11.1, SD 3.5) were interviewed. Two thirds were boys (*n* = 24; 66.7%) and one third were girls (*n* = 12; 33.3%). Most students attended mainstream educational settings (*n* = 31; 86.1%, often with integration aids), with others attending disability-specific settings (*n* = 3; 8.3%), or support classes within mainstream schools (*n* = 1; 2.8%). Students were from a variety of cultural backgrounds, including Chinese (*n* = 11; 30.6%), Vietnamese (*n* = 9; 25.0%) Somali (*n* = 4; 11.1%), Lebanese (*n* = 2; 5.6%), and White Australian (*n* = 10; 27.8%). The participating parents of the 10 children (27.8%) from White Australian background identified as Autistic. Table 1 presents summary participant demographic information.

**Table 1.** Summary Demographic Information for Study Participants.

	Chinese *n* = 11	White Australian/Autistic Parents *n* = 10	Vietnamese *n* = 9	Somali *n* = 4	Lebanese *n* = 2	Total *n* = 36
Age (years) M(SD) range	9.1 (2.7) 4–13	11.0 (3.4) 5–16	13.1 (2.9) 9–17	12.8 (4.8) 6–17	13.0 (0.1) 8–18	11.1 (3.5), 4–18
Gender						
Boys	7 (63.6%)	5 (50.0%)	7 (77.8%)	3 (75.0%)	2 (100.0%)	24 (66.7%)
Girls	4 (36.4%)	5 (50.0%)	2 (22.2%)	1 (25.0%)	-	12 (33.3%)
Educational setting						
Preschool	1 (9.1%)	1 (10.0%)	-	-	-	2 (5.6%)
Primary	9 (81.8%)	5 (50.0%)	3 (33.3%)	1 (25.0%)	1 (50.0%)	19 (52.8%)
Secondary	1 (9.1%)	4 (40.0%)	6 (66.6%)	3 (75.0%)	-	14 (38.9%)
VCAL	-	-	-	-	1 (50.0%)	1 (2.8%)
Type						
Mainstream	8 (72.7%)	10 (100.0%)	9 (100.0%)	2^b^ (50.0%)	2 (50.0%)	31 (86.1%)
Specialist disability	1 (9.1%)	-	-	2 (50.0%)	-	3 (8.3%)
Support class	1 (9.1%)	-	-	-	-	1 (2.8%)
Dual schooling^a^	1 (9.1%)	-	-	-	-	1 (2.8%)

*Notes*: VCAL = Victorian Certificate of Applied Learning. ^a^Enrolled in both mainstream and specialist schools. ^b^One participant recently moved from a specialist disability setting.

### Procedure

Semi-structured student interviews were conducted between May and December, 2021, by two clinical researchers with extensive experience working with Autistic children and young people. Given local restrictions due to the COVID-19 global pandemic, interviews were largely conducted via Zoom (*n* = 33; 92%) or over the telephone (*n* = 2; 5.5%), with one interview conducted in person. Consent was provided to record interviews. Students were asked about their school, including subjects, teachers, strategies that helped them to learn, their friends and how they were treated by others. Key interview questions were shared with parents and students beforehand. Where appropriate, visual aids (e.g., picture cards of school subjects/classroom activities and visual ratings scales) were used to support students’ understanding and facilitate their responses. These aids provided structure to the interview process (Lewis et al., 2008) and allowed for verbal and non-verbal communication by students, with parental support, to optimize their ability to participate (Aldridge, 2007; Hummerstone & Parsons, 2020).

To prompt further discussion beyond the interview questions, students were given a Drawing the Ideal School activity (see Gray et al., 2023 which built on Moran (2001) Drawing the Ideal Self technique). The process was followed as per Gray et al. (2023). In this activity, students were asked to draw two pictures—one of their non-ideal and another of their ideal school—naming elements of each classroom, and describing the other pupils and teachers. Task instructions were adapted to suit child age and language level (i.e., asking students to draw a school they would “really like to go to” or “not like to go to”). If students did not want to complete the activity during the interview, parents were send instructions and asked to attempt the activity at home. Such use of visual (or pictorial) methods can shift control of the interview process to the interviewee, reducing social and language demands and thereby allowing an unlimited range of responses (Lewis & Porter, 2004) and richer understanding of how students make sense of their educational experiences (Humphrey & Lewis, 2008). The drawings were not analyzed but rather used to elicit further description from students, captured in the interview transcripts. Interview recordings were subsequently transcribed verbatim using a transcription service. See Supplementary Materials for interview questions, including Drawing the Ideal School activity for parents.

### Data Analysis

The interview data was analyzed using **s**emantic-based reflexive thematic analysis within a “Big Q” qualitative framework, as outlined by Braun and Clarke (2006; 2019). An inductive approach was used to identify patterned meanings in the dataset (i.e., without integrating researcher preconceptions or pre-existing coding schemes) within an essentialist framework, whereby our goal was to recount the experiences, meanings, and reality of the Autistic student participants. Our team members brought a range of positionalities to bear on the analysis, including professional backgrounds in speech and language therapy (JS), psychology (GC, EP, ASR, WL, KH), nursing (GH), anthropology (RL), and education (PD, ED, MH, RL, EP, SR), as well as positionalities as Autistic researchers and advocates (WL, GH and MH) and parents of Autistic children (WL, GH, MH and RL).

To begin the analysis, two senior researchers and a student researcher immersed themselves in the data, taking notes on striking and recurring observations, and using NVivo to apply codes line-by-line to each transcript. A draft thematic map was generated. This showed potential themes and subthemes, with relevant quotes shared for discussion and refinement with other members of the research team, and with one Autistic student research participant, prior to finalization.

## Results

**Figure 1.**
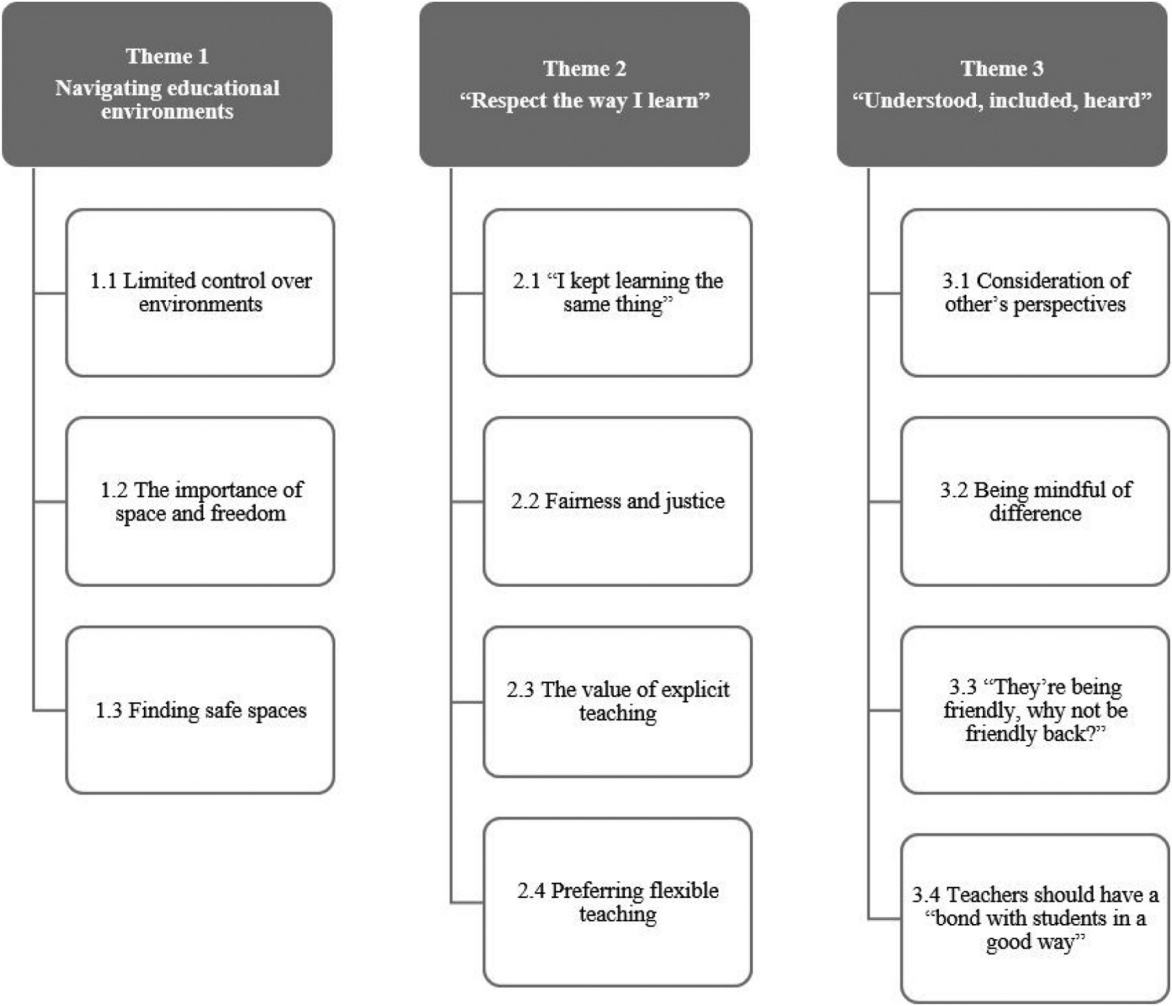
Key Themes and Subthemes Generated From Analysis of Autistic Students' Education Experiences.

We identified three broad themes, depicted in Figure 1. To highlight breadth and depth of responses, illustrative quotes are attributed to participants signaled by participant ethnicity (C = Chinese, V = Vietnamese, W = White Australian, S = Somali and L = Lebanese), a randomly-generated number (e.g., [17]) followed by education setting (M = mainstream, S = specialist setting and SC = support class, V = VCAL) and age.

### Theme 1. Navigating Educational Environments

**Figure 2.**
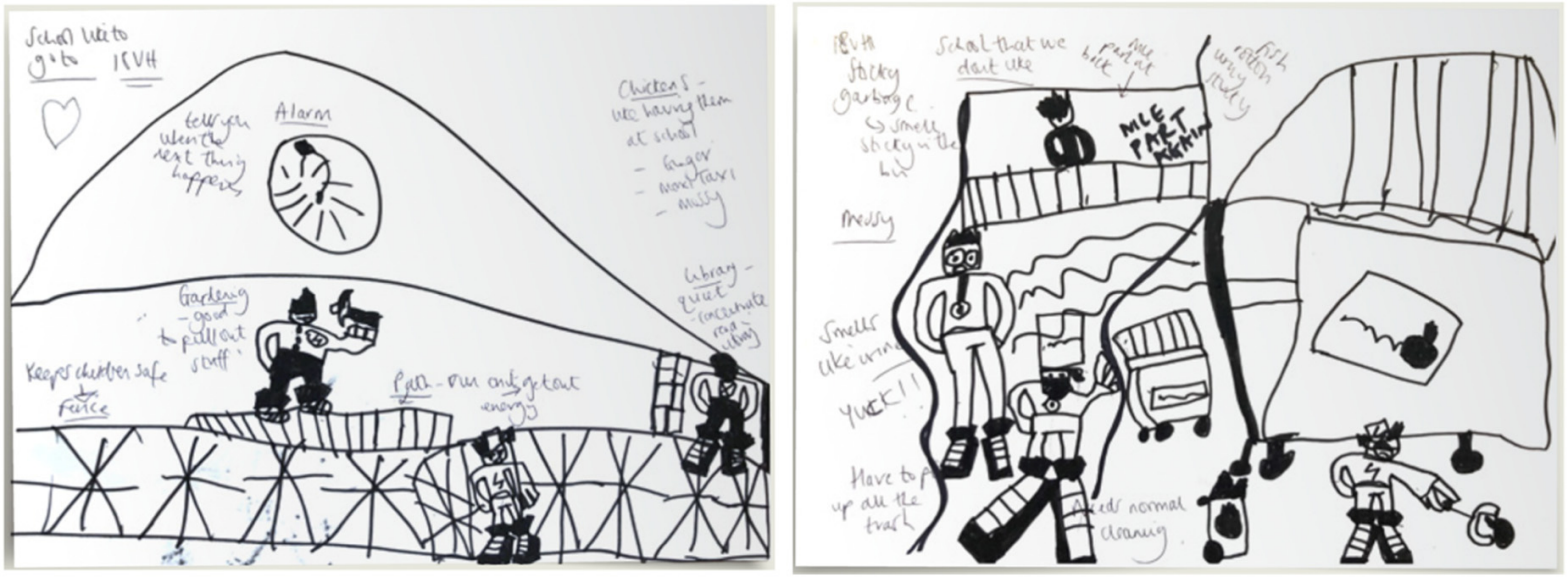
Ideal (Left) and Non-Ideal (Right) School Drawings by a 9-year-old Vietnamese Autistic Student Attending a Mainstream Australian Primary School, With Handwritten Notes Made by the Interviewing Researcher as the Student Described his Drawings.

On the first theme related to the school environment, students expressed that educational institutions were places for studying: “Learning's important for me” [W015_M_15]; “At school you want to do the best you can” [V006_M_16]. Generally, students said they “enjoy[ed] going to school” [C046_S_13], with one commenting that home learning during the COVID-19 pandemic was “okay” but that he would “rather be at school” [W003_M_11]. Despite this, students found school challenging as they had *limited control over environments* (subtheme 1.1). Students described settings as “noisy” [V019_M_8], “cramped” [V002_M_17], and “really hot and stuffy” [W020_M_14] and spoke about the “sensory nightmare” of “hand dryers” and “chalk dust [that] goes everywhere” [W009_M_10]. The journey to school was also overwhelming for some: “When there's so many people in the bus, it['s] so awkward” [S035_S_7], and many described feeling overwhelmed by teachers who “shout a lot” [W011_M_9], “yell and talk aggressively” [V053_M_13], and “just scream at [them]” [C010_M_11]. One student reflected that it was not necessarily being reprimanded that was difficult, but the sensory aspect of being spoken to loudly: “I don’t like getting yelled at because it's very loud” [W014_M_10]. Students reported on the real physical impact of the sensory environments they encountered: “I don’t like a noisy space. It makes me have a headache and when it's noisier, it gets worse and hurts a lot” [V019_M_8]. One set of drawings (Figure 2) reflected students’ sensory issues, with the Non-ideal School described as messy, smelly and sticky.

Students spoke of how classroom design impacted their learning: “Open classrooms… [are] very annoying, because you can hear other teachers. If another teacher's mad in another classroom, you can hear them” [V006_M_16]. An expectation to remain seated for long periods of time was also challenging: “Just sitting there for 15 min and not getting up is not my type” [C010_M_11]; “I hate sitting down and just waiting because I fidget” [W011_M_9]. The impact of dealing with such environments across the day meant students often needed to decompress at home: “When I get home from school, I’m always exhausted so I’m always a lot quieter… I feel comfortable just being quiet” [W020_M_14].

Since students often felt unable to control their broader environments, they unsurprisingly highlighted *the importance of space and freedom* (subtheme 1.2) within the day. One student liked “to listen to music during class time” [C010_M_11], while another used “headphones [to calm down]” [V019_M_8]. Another stated that he enjoyed and liked to be able to “make loud noises” [V018_M_9] himself. Students also valued time away from the confines of the classroom, including appreciating outside play to go “running around, chasing everyone” [C034_M_7] and using up “lots of energy” [V018_M_9]. One student described how he could “handle” his physical education class “because it's in [a] really open space no matter where we are, so I can be okay” [W001_M_7]. Another remarked that “having fresh air” [W003_M_11] helped them to learn.

One important way students from across backgrounds expressed being able to control their sensory environments was by *finding safe spaces* (subtheme 1.3). They valued having “rest places, quiet places” [V019_M_8], or “a place [to] sit and relax” [C039_M_9]. They wanted places to retreat to when needed: “I just ask the teachers if I can go to the toilet and then I have a little time when I feel better” [W011_M_9]. Many students identified the library as a safe space: “I like to be quiet in the library, so no-one can get distracted” [V018_M_9]; “I found this perfect little nook that's sandwiched between a little one of those turning book things, so no one can really disturb me there” [W009_M_10]. Students identified other unique safe places too: “My favourite place in school is with my friends, underneath our ramp… there's no one there to bother us and it's like a secret area” [C010_M_11]; “There's a short tree… we jump onto the tree, and just sit there, and talk about stuff… it's not as crowded as the playground where everyone is screaming in my ears” [L060_M_8]. Alongside depicting sensory issues in a Non-ideal School, the illustrations above (see Figure 2) included a path to run and “get energy out” and a library to provide a quiet space to concentrate, in this student's Ideal School.

### Theme 2. “Respect The Way I Learn”

The second theme relates to the methods and practices of teaching. Firstly, many students—especially those from ethnic minority backgrounds—reported school to be insufficiently challenging: “*I kept learning the same thing”* [S028_M_17] (subtheme 2.1). Some felt schoolwork was a “bit easy” [V053_M_13] and that they could complete tasks “very easily” [C048_M_11]. They bemoaned the repetitiveness of their lessons: “I’m in the lower math class and it's a bit too easy so it gets boring for me. And spelling, I just don’t really find it interesting and it's a bit repetitive”. Another student reflected: “There's no fun repeating the same things I know already*”* [S028_M_17]. Secondly, students disliked “when [the teacher] made [work] easier” [C062_M_10]. One student said: “I’d like a bit more [homework], just enough to keep me busy” [V002_M_17]. They described mediocre expectations, both for their behavioral conduct and academic achievement: “As long as you are doing average… as long as you’re not distracting other students, it's fine” [V006_M_16]. Despite liking school overall, students who felt unchallenged in the classroom, expressed that “sometimes, school life [was] quite boring” [C046_S_13].

Yet, students across backgrounds were also frustrated by a perceived lack of *fairness and justice* (subtheme 2.2) in education settings. They sometimes expressed irritation when peers did not follow school rules: “Once it was so loud, [the teacher] went, ‘shush, shush’ and no one stopped” [W001_M_7]; “[The teacher] is saying to be quiet, but they keep on doing it” [S037_M_13]. One student complained that although she frequently raised her hand to answer questions, her teacher “asks the kids who aren’t putting their hands up and who don’t usually put their hands up”, describing this as “a bit frustrating” [W014_M_10]. Another explained being “separated” from other students for being “a chatterbox”, while “many [others] chat more and they’re not separated” [L060_M_8]. Another similarly recalled not being allowed to play games on his electronic device during recess yet “some people do that” but are not stopped, because the teacher had not “caught anyone” [C062_M_10].

It was especially frustrating for students when they experienced the consequences of others not following the rules: “The only thing I don’t like about my classroom is that there are very naughty boys in my classroom, and they make us stay back at recess all the time. And it's not fair for us” [L060_M_8]. The injustice that students should be penalized for others’ actions led one student to assert: “I don’t like collective punishment” [W015_M_15]. Likewise, students were perplexed when they were reprimanded at school and were unsure why: “[The teacher], she wrote up on the board that I was standing for five minutes and I didn’t really understand what I did wrong. So I asked, ‘What did I do wrong?’ Then she just yelled at me to be quiet” [W014_M_10].

Regarding taught content and learning activities, students highlighted *the value of explicit teaching* (subtheme 2.3). They complained about teachers “not explain[ing] things properly” [V044_M_13] or when instructions appeared contradictory—“Do this but don’t do this” [W014_M_10]. These opaque instructions meant students got “really confused and [didn’t] know what to do” [C027_M_12] or “understand what's being taught” [V002_M_17]. Excessive information was similarly unhelpful: “[Teachers are] giving me too much information… I get a headache. I just can’t handle that much information… feel like I’m going to pass out” [W077_M_16]. Frustration also followed when students requested clarification, but received no additional advice or information to support their understanding: “I remember I was asking a teacher about this Maths stuff, and basically what happened was they explained it, but they didn’t really explain it too well. So, I asked again, and they just did the same thing” [W015_M_15]. One student described frequent “miscommunication” when they asked for help, as teachers “don’t fully understand what I’ve been asking for” [W020_M_14].

Conversely, students reported that helpful teachers provided “a clear outline of what to expect each week” [V002_M_17] and useful strategies to ensure students can complete their tasks: “Some of my teachers are very organised on [Microsoft] OneNote… I know exactly what happened in class. So, if I didn’t get my work done, I can just look at it when I get home and get it all done” [W020_M_14]. Students appreciated when teachers “put in lots of detail” [S037_M_13] and “explain everything” [V044_M_13] “over and over, if needed” [W009_M_10]. Students likewise valued teachers who were explicit about letting them “know *when* [they] did well” [V025_M_12] and proactively explained *how* to do well:

One of my favourite teachers… she's extremely organised and she tells things how it is. So, if I went up to her with my assignment and asked for any pointers of how to improve it, she would tell me that this part isn’t so good and I need to add this [W020_M_14].

Explanations were especially useful for more subjective lessons, such as English: “the way English is marked is very subjective… there's multiple answers, and ways you can answer in an English question, like an essay” [V006_M_16]. They reflected on the complexity of English lessons: “We have to understand text, and I have to try to understand their meaning” [V053_M_13]. Since students appreciated being challenged at school, they liked it when teachers clarified instructions without lowering expectations: “My English teacher, we were doing this essay, and she made it clear enough for us to understand… but not too easy” [S037_M_13].

Students also stated *preferring flexible teaching styles* (subtheme 2.4) over more authoritarian ones. When students described unhelpful teachers, this included those who were “very, very strict” [C070_M_9] and perceived to want students’ “submission, attention” [L004_V_18]. Students did not want a teacher who “doesn’t really let [them] have any fun… just tells [them] to do work all the time” [C010_M_11]. Another commented that he disliked a particular teacher “because he's very strict and he is an authoritarian” [W015_M_15]. Students also described dislike for “short deadlines” [W015_M_15] as when they “don’t get much time… that doesn’t really help [me] learn” [W011_M_9]. Stricter approaches to teaching were also more likely to pressure students into doing things they were uncomfortable with in the classroom. One student recalled being made to participate in dance at school: “[The teacher] forced me to do stuff… she was like, ‘it's too bad. You’re going to have to do it’. Even though I didn’t want to do it, and I felt really uncomfortable” [W009_M_10]. One student explained how a teacher “forced [her] to eat fruit” [S035_S_7]. One student stated: “If you release that strictness, students will be more willing to be more out of the box” [L004_V_18].

**Figure 3.**
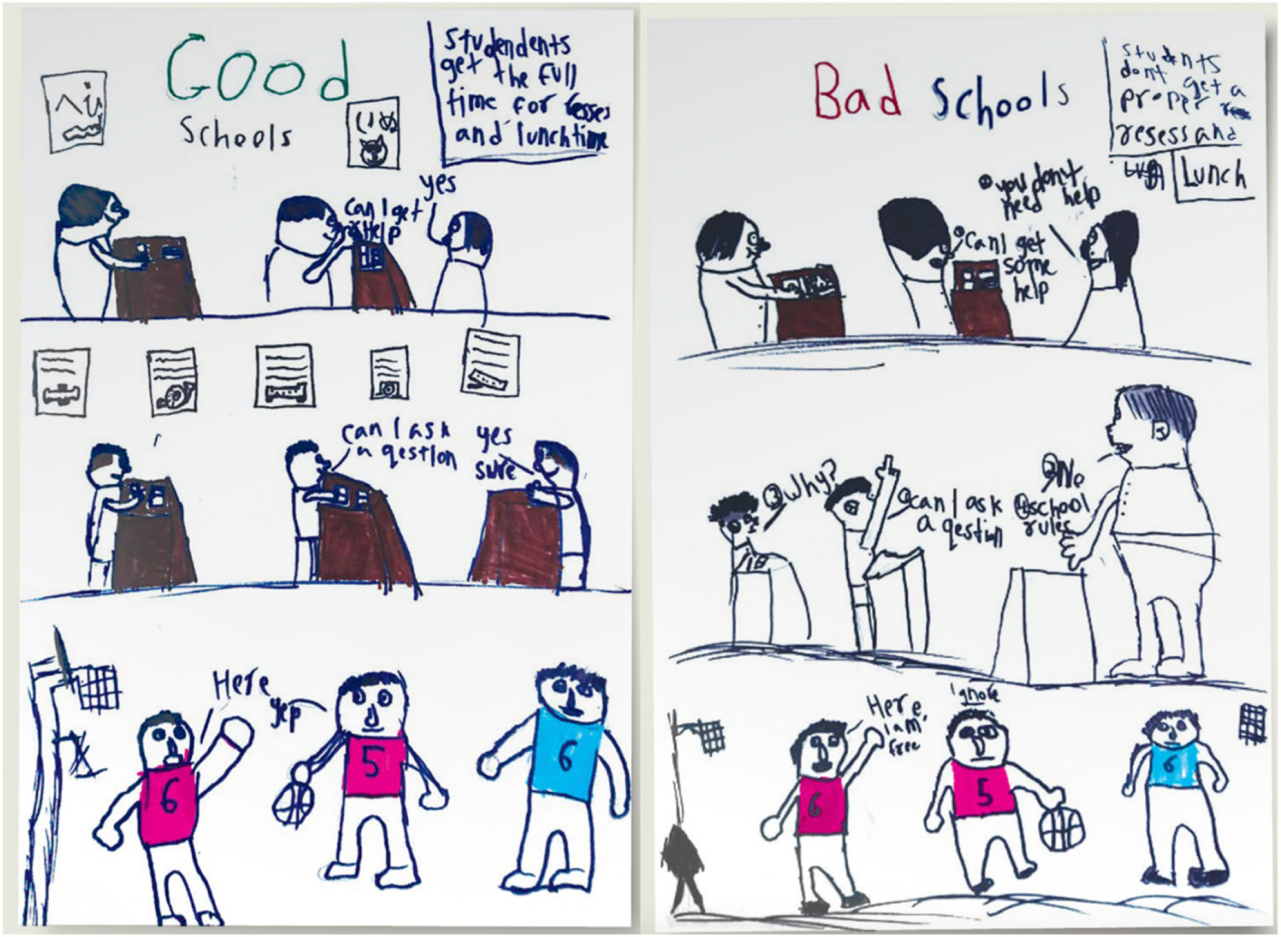
The Ideal (Left) and Not-Ideal (Right) School Drawings of a 13-year-old Somali Autistic Student Educated in a Mainstream Australian Secondary School.

Conversely, students valued teachers who were accommodating and adaptable. Figure 3 depicts one student's Ideal School where students can ask questions and access help, compared to his Non-ideal School where teachers dismiss students’ needs. Students described liking the flexibility to decide where they worked and what respite they required: “[The teacher] lets me take breaks if I need them and I can go into the space in between the two classrooms called the bubble and just do my work there” [W014_M_10]. They also liked being able to go to the bathroom “anytime [they] need[ed] to” [C_001_M_4]. They also valued teachers who were flexible with timelines for completing work: “If you can’t do the work that day, they just ask you, get it done later” [W020_M_14]. One student described that they appreciated a specific teacher who “just gave me the freedom. I don’t like to do homework. I don’t like to do writing. But [teacher] didn’t … push me to do the writing, or do all sorts of homework” [C046_S_13].

### Theme 3. “Understood, Included, Heard”

The final theme centers on the interpersonal aspects of school life. It was clear that Autistic students often gave conscious *consideration of others’ perspectives* (subtheme 3.1). Students adapted their behavior to accommodate others’ needs, including one explaining how he “obviously” did not “act the same at school and home, because there are friends in school and at home there are family members, so you have to treat them differently” [S037_M_13]. Another similarly described considering his teachers’ and peers’ perspectives in the classroom: “I don’t like asking for help, because I feel like they already have enough on their plate, with everyone else in the classroom… I don’t want to be taking up their time where other kids could be using it” [W009_M_10].

**Figure 4.**
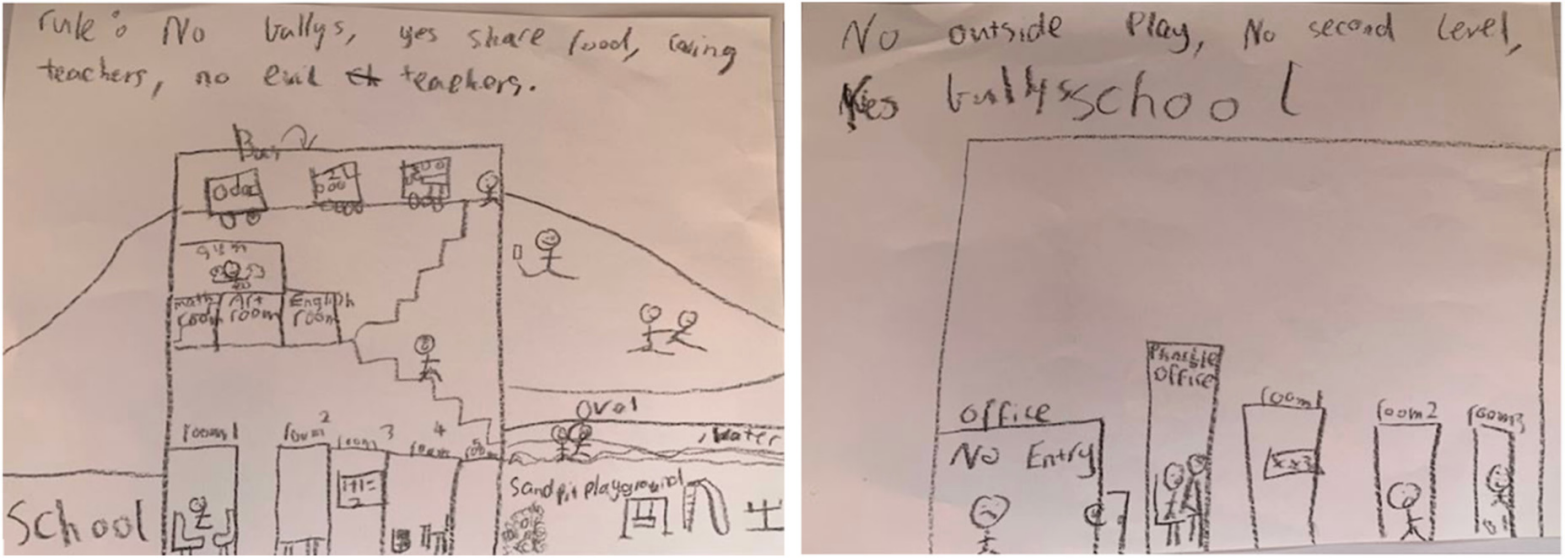
The Ideal (Left) and Non-Ideal (Right) School Drawings by a 9-year-old Chinese Autistic Student in Australian Mainstream Primary Schooling.

Consideration of others’ perspectives extended to students *being mindful of difference* (subtheme 3.2)—this included students from all backgrounds: “I’m born in the same country, in Australia, but I’m Vietnamese” [V053_M_13]; “[My friends are] short, Asian, Chinese, black hair - kind of look like me” [C048_M_11]. Students were aware of discrimination; of how people can “criticise and judge depending on if [people] have disabilities, they’re LGBTQ, they’re not that smart… their skin colour, gender” [W014_M_10]. Some described having witnessed or experiencing discrimination: “[Peer] was so fricking rude. He didn’t like people like girls, like brown girls… he likes to bully people” [S035_S_7]. Students did not want intolerant schools where “people like to start an argument and are racist” [S028_M_17], where people told “racist jokes” [L004_V_18] or which were “racist, strict, [with] a lot of bullies” [W005]. Rather, they reported wanting schools that were “multi-cultural, that is a big must” [W005] and where differences are valued. They also wanted schools to accommodate differences, such as including “wheelchair lifts and ramps” [W014_M_10]. Figure 4 shows an Ideal School drawing, with children playing together and sharing food, compared to a Non-ideal School with bullies.

Students believed in being welcoming towards others: “*They’re being friendly, why not be friendly back?”* [W015_M_15] (subtheme 3.3). Many students reported having friends: “I have quite a number of friends; some in my class, some I hang out with online” [V002_M_17]. Even if students did not report having many intimate friendships, they felt they were not *disliked* by others: “I’m not super popular but I’m not really unpopular either” [W014_M_10]; “Well, I get along with everyone, I’m not really close with everyone but I get along with people fine” [W020_M_14]. Their perception of being “normally treated pretty well” [W015_M_15] by peers was seemingly connected to the way they “treated [others] with [the] respect that they deserve” [V002_M_17]. Nevertheless, they were aware of qualities that made some peers *less* popular at school; “arguing… start[ing] conflict” [S028_M_17] or “act[ing] kind of aggressive” [V025_M_12]. Even when peers exhibited these behaviors, however, Autistic students felt sympathy and understanding were the best approaches:

There was one kid I know who wasn’t a friend, but I just knew him. He was very aggressive… just say one thing that annoyed him, and he would actually try to start a fight…If someone did understand that kid and why he's doing it, maybe they could help him to not be as stressed and make it so he could take a loss [L004_V_18].

Students similarly valued respectful, caring relationships with teachers, and emphasized that *teachers should have a “bond with the students, in a good way”* [V002_M_17] (subtheme 2.5). They felt that teachers should “treat [students] more like an equal and not like a subordinate” [W015_M_15] and be “patient and kind and understanding” [W009_M_10] and “really nice to [them]” [W011_M_9]. But they also sought teachers who were “humorous” [W009_M_10] and “play[ed] with them” [C046_S_13]. One kindergarten student described enjoying playtimes because his teacher “pretends she's a monster, and she chases everyone” [C034_M_7].

Students clearly valued teachers who cared about them as individuals. One student described a teacher who could “read [him]” so would know when he had not understood something and would just “confirm” what to do, which “really helped” [W015_M_15]. Another explained why her Grade 2 teacher had been her “favourite so far… I just feel like she understood me, and knew what I needed” [W009_M_10]. Individualized supports were described as key to a good education experience. Students wanted teachers who “sat next to [them] and helped” [C034_M_7]. One student clearly summarized the individual supports his favourite teacher provided:

Every time I have meltdowns or… I struggle, [teacher] always sits next to me, and helps me out and gets me through this because he knows I could do better than just giving up and sitting around doing nothing… He's very kind; I like him a lot. Every time I have issues or any questions he always responds in a respectful way and gives up his time for me. [W077_M_16]

## Discussion

We report on the education experiences of Australian Autistic students of varied ages, cultural backgrounds, and educational settings, including their environmental, pedagogical, and relational experiences. These findings are discussed in detail below.

### Towards Enabling School Environments and Practices

A number of studies have previously reported that Autistic students find many aspects of school environments are not adapted to their needs and preferences (Friedlander, 2010; Howe & Stagg, 2016; Saggers, 2015). This study reinforces those findings. Similarly, accounts of how inhospitable environments lead to sensory overwhelm causing Autistic students to feel physically and mentally unwell have also been previously reported (see Birkett et al., 2022; Hasson et al., 2022). Although students in this study felt that many aspects of schools were ill-suited for them, they still described preferring being educated in person rather than online (as many children were required to do during the COVID pandemic). These findings—articulated here for the first time by a broad range of Autistic students of different ages, abilities, settings, and backgrounds—reinforce how classroom design and education practices must consider the needs of *all* students for Autistic students to thrive.

Interestingly, the desire for space and freedom, including quiet places to relax, was more often commented on by younger learners, possibly as they have less agency and flexibility within their school days as compared to those in secondary school. Many suggestions from our participants—such as the use of headphones in class, or spaces to retreat to prevent or manage sensory/social overwhelm—could be “quick wins” for schools wanting to foster safer, more secure settings for Autistic learners, especially younger students who may have less autonomy within their educational environments.

Past studies have recounted how Autistic students can struggle with academic demands (Carrington & Graham, 2001; Gray et al., 2023). Interestingly, however, we found the opposite here; many students across our diverse sample accessing mainstream and specialist education settings, wanted schoolwork to be *more* challenging. Other Autistic youth, with varying language and cognitive abilities, have described education experiences as a barrier to achieving their future goals and complained of “being treated like an idiot” at school (Tesfaye et al., 2023, p. 1149). Autistic adults schooled in specialist classrooms within mainstream settings have likewise reported being insufficiently challenged, academically (Croydon et al., 2019; Zakai–Mashiach, 2023). Plausibly, educators may underestimate the academic abilities or education ambitions of Autistic students, especially those educated within specialist settings or those with higher support needs. Still, feeling insufficiently challenged was reported by our participants *across* both mainstream and specialist settings alike. This finding may be driven by the presumption of limited competence held by schools which is rooted within deficit-based models of autism (see Biklen, 2020; Jorgensen, 2018).

For the current study participants, such accounts might also have been compounded by the fact that most were from minority-ethnic backgrounds groups. Indeed, lower academic expectations and being insufficiently challenged were more commonly reported by ethnically diverse students compared to those from Australian backgrounds. Extant studies have likewise reported low academic expectations toward non-Autistic Black (Malone et al., 2023) and refugee students (Ziaian et al., 2020). Parents from minority-ethnic backgrounds have likewise described teachers’ low educational expectations for their Autistic children (Smith, Rabba, Ali et al., 2023), expectations which can be seen in contrast to high parental academic expectations in some cultures (see Qu et al., 2020; Smith, Rabba, Cong et al., 2023).

Our data indicates that expectations of students may again be intersecting with their ethnic backgrounds. Nevertheless, whether our Autistic participants felt insufficiently academically challenged due to their Autistic identity, their particular educational settings, their minority-ethnic background *and*/*or a combination of these*, students keenly felt that schools should be places where they could learn and be tested. We too endorse high expectations for all Autistic students, with a focus on ensuring students from minority-ethnic backgrounds are challenged and extended in all educational settings.

Aligning with past research (Saggers, 2015; Saggers et al., 2012), students clearly valued teachers who practiced structured flexibility and provided explicit instruction—especially secondary-aged students for whom organizational and academic demands are greater. The benefits of individualized, systematic instruction within structured learning environments and pedagogical practices has been recommended for over two decades (Iovannone et al., 2003). What was noteworthy in this study was students’ explicit expressed dislike for, and sometimes fear of, erratic authoritarian teaching styles and unpredictable school environments; an experience that can contribute to making formal education settings untenable for some Autistic students. Indeed, Heyworth et al. (2021) found that many Autistic students they interviewed reported positive home-learning experiences in the context of the COVID-19 pandemic, in part because they or their parents could proactively manage the home-learning environment to foster predictability, thereby preventing the overwhelm so often associated with classroom schooling. It is important that teachers aim to embed as much certainty into Autistic students’ educational environments as possible in order to lay a solid foundation for student learning and ensure students feel safe and supported within kindergartens and schools.

## Relationships with Peers and Teachers

The Autistic students we interviewed valued teachers who showed mutual respect, fun and responsiveness to their individuality and support needs. These findings echo past research describing active listening, responsiveness, connection, and light-heartedness as desirable educator qualities (Gulec-Aslan et al., 2013; Humphrey & Lewis, 2008). Past researchers have articulated how an “ethics of care” is integral to the educational experience of Autistic students, especially for those marginalized learners at greatest need of feeling heard and understood (see Fernandes, 2019; Noddings, 2012 for details). We concur that the most effective learning for Autistic students is predicated on trusting student-teacher relationships built on teachers understanding the unique strengths, needs, personalities, and identities of each individual (Heyworth et al., 2021).

Much research has reported how Autistic students struggle to feel included socially at school, and experience teasing and bullying by peers (Gray et al., 2023; Humphrey & Lewis, 2008; Tesfaye et al., 2023). Fortunately, our participants described these experiences as uncommon; rather, they described being liked and respected by peers. Perhaps our heterogeneous group of Autistic students allowed more positive aspects to come to the fore. Past research has largely focused on a narrow group of Autistic learners, namely those who are White, male and mainstream educated. Ainscow (2022) argued that focusing on specific types of learners can perpetuate deficit-based views. We suggest that participant homogeneity within extant research may have contributed to the underreporting of more positive aspects of the educational experience (Lynam et al., 2024).

Another possible reason for why our students often described being liked and respected by peers may be because they treated others with fairness and respect. Past studies have reported a strong sense of justice among Autistic adults (Dempsey et al., 2020; Greenberg et al., 2024) and treating peers fairly is also driven by this stance. Importantly, all students in this study were from marginalized backgrounds (whether through neurotype and/or minority-ethnic community status), and so being mindful and accepting of difference, and seeking to treat others fairly, may be further ingrained than among other participant samples. Overall, it is heartening that negative social experiences were uncommon in our study. This hopefully indicates a shift towards greater acceptance and more meaningful inclusion of Autistic students within education settings.

## Limitations and Future Directions

Our study had several limitations. First, although we used multimodal methods to elicit Autistic students’ views on their education experiences, we were unable to reach children and young people who were non- or semi-speaking. Further, our interviews were conducted over Zoom during the pandemic. While there are benefits to online interviews (i.e., participants feeling more comfortable in their home environments and being able to access a diverse range of participants across locations; Boland et al., 2022; Oliffe et al., 2021), face-to-face interactions may have been more beneficial for some Autistic participants to build rapport, trust, and connection (Ritzman & Subramanian, 2024). Future research should attempt to gather novel information from both younger children (i.e., preschoolers) and non- or semi-speaking children, exploring methods suited to this purpose.

Further, we decided not to ask children directly about how their minority-ethnic backgrounds might intersect with their autism in determining their school experience as we believed such questions could have overly influenced their responses. Although some children did spontaneously discuss their cultural backgrounds, especially around interactions with peers, this omission may have limited our understanding of the unique intersectional educational experiences of these Autistic students. We similarly did not directly elicit students’ views and experiences of pandemic-related lockdowns which may have been useful for contextualizing our findings. Finally, gathering educators’ perspectives, in particular about their training needs to support Autistic students—including those from marginalized groups—will be important future research.

## Conclusion

This innovative study explored the educational experiences of Autistic students from diverse ages, different cultural backgrounds, and educational settings. Thoughtful school, classroom and curriculum design, high academic expectations and feeling valued as individuals were all reported as essential for an effective learning experience. Our findings have highlighted that many aspects of the educational environment that could promote Autistic students’ learning are modifiable, including access to safe spaces and sensory supports, explicit teaching practices, predictable classroom routines, and teachers’ openness to listening to and working with their individual students. We hope these findings inform how we can enhance learning, social and wellbeing outcomes for Autistic students across all ages, abilities, and backgrounds.

## Supplemental Material

sj-docx-1-dli-10.1177_23969415251377973 - Supplemental material for “I Just Feel Like the Teacher Understood Me, and She Knew What I Needed”: School Experiences of Autistic Students from Diverse Backgrounds Supplemental material, sj-docx-1-dli-10.1177_23969415251377973 for “I Just Feel Like the Teacher Understood Me, and She Knew What I Needed”: School Experiences of Autistic Students from Diverse Backgrounds by Jodie Smith, Aspasia Stacey Rabba, Georgia Coverdale, Poulomee Datta, Gabrielle Hall, Melanie Heyworth, Kristelle Hudry, Wenn Lawson, Rozanna Lilley and Elizabeth Pellicano in Autism & Developmental Language Impairments

sj-docx-2-dli-10.1177_23969415251377973 - Supplemental material for “I Just Feel Like the Teacher Understood Me, and She Knew What I Needed”: School Experiences of Autistic Students from Diverse Backgrounds Supplemental material, sj-docx-2-dli-10.1177_23969415251377973 for “I Just Feel Like the Teacher Understood Me, and She Knew What I Needed”: School Experiences of Autistic Students from Diverse Backgrounds by Jodie Smith, Aspasia Stacey Rabba, Georgia Coverdale, Poulomee Datta, Gabrielle Hall, Melanie Heyworth, Kristelle Hudry, Wenn Lawson, Rozanna Lilley and Elizabeth Pellicano in Autism & Developmental Language Impairments
