# Supplementary material for: “I Just Feel Like the Teacher Understood Me, and She Knew What I Needed”: School Experiences of Autistic Students from Diverse Backgrounds
Source: Autism Dev Lang Impair. 2025 Oct 15;10:23969415251377973. doi: 10.1177/23969415251377973 (PMC12535640; doi:10.1177/23969415251377973)
Supplement: sj-docx-1-dli-10.1177_23969415251377973 - Supplemental material for “I Just Feel Like the Teacher Understood Me, and She Knew What I Needed”: School Experiences of Autistic Students from Diverse Backgrounds [file sj-docx-1-dli-10.1177_23969415251377973.docx]

**Students’ or young peoples’ experiences of school**

Hi, my name’s XXX, and I’m a researcher on this project.

Thank you for agreeing to help out with our project. I’m going to tell you a bit more about what this is. We want to find out what life at school is like for you. To do this, we’re going to ask you some questions about what bits you like and don’t like about school, and your friends, and what things you would like to do more of.

I have 6 different categories I’d like to talk about – (1) you and your school, (2) your school subjects, (3) learning at school, (4) choices you can make at school, (5) people at your school, and (6) your ideal school. You can choose to talk about these topics in any order that you like.

There are no “right” or “wrong” answers in any of this; we just want to know what you think and feel about things. All the answers will be kept between you, me, and the other researchers on the project. And we won’t use your name in anything we produce from the project.

We’ll be talking about a few different topics that might bring up some different feelings. I hope that you are okay talking about these things. But if at any time you decide that you don’t want to talk anymore then please tell me and we can stop.

I have a picture of a traffic light so you can show me when you’re happy to talk about things (green), when you need a break (orange) or when you want to stop (red). Do you want to use those? You can also just tell me if you would like to take a break or stop.

As you know, we would like to record this interview so that it can be written up later. Is that still ok? Great – thank you.

Do you have any questions, before we start? ......... Ok, let’s get started!

**Start the Interview**

**BACKGROUND**

**What is your favourite thing [to do, think about, learn about, talk about, spend time on] in the world?**

- *I hear you like […]. I can see you like […]. Can you tell me about that?*

**Tell me a bit about your family.**

- *Who lives with you at home?*
- *What about pets?*

**Tell me a bit about your school. What do you like about school?**

- *What school do you go to?*
- *How long have you been going to this school?*

**_______________________________________________________________________________**

**LEARNING AT SCHOOL**

**Primary questions: What are your favourite subjects? What are the ones you don’t like so much? Why? Are there things that make it easy or hard for you to learn at school? Is there something that your teacher does that helps you to learn? Is there something that your teacher does that makes it harder to learn?**

**Probe questions:**

*In the classroom:*

1. Do you get to work with your friends?
2. Do you have homework? How much? Who helps with this?
3. Do you get the help you need at school? Who helps you? How do you ask for help if you need it?

*Around school:*

1. What do you do at lunch and breaktimes?
2. Are there clubs and activities you can join in with at lunchtimes? What about after school?
3. Do you do sports activities? Do you get to go on excursions, camps, etc.?
4. Is moving around school easy? How do you find moving between classrooms, out to the playground? Do you know where you need to be and at what time? How do you find assemblies?

_________________________________________________________________________________

**HAVING YOUR SAY**

**Primary questions: Do people ask you about what makes school good or not-so-good for you. Do you get any choices about what you do at school and how you do it? And what kind of help you get?**

Probe questions:

1. How much choice do you have about:
   1. what you learn every day [e.g., during story time]?
   2. how you show what you’ve learned?
   3. who you sit next to?
   4. who supports you? Who helps me? Do you feel helped?
   5. what clubs/activities/breaktimes you go to?

**_________________________________________________________________________________**

**PEOPLE AT SCHOOL**

**Primary questions: Do you have people you like to spend time or play with at school? If yes, can you tell me about them? What are their names, and what do you normally like doing together? If no, do you have people you share an interest with?**

**Tell me about your teachers. Who is your favourite teacher? What do you like about them? Do you have teachers you definitely *don’t* like? Why?**

**Who do you go to at school when you need help or feel worried/stressed?**

**Probe questions:**

1. Would you like more friends? Do you think you need help making/keeping friends? Who can you turn to for help with this?
2. How would you describe yourself or how do you think of yourself? Are you similar or different to your friends or other children you go to school with? If so, how?
3. What do the adults/teachers in school think of you? How do they interact with you? Does it change between teachers?

_________________________________________________________________________________

**STAYING SAFE**

**Primary question: How are people treated in school? How are *you* treated in school?** **Do you act differently at home than when you’re at school? Why do you think you do that?**

Probe questions:

1. Does the school say anything about how to treat people in the school?
2. Do you feel safe at school? What about at lunchtimes/breaks? Do other children or teachers bother you? Who makes you feel safe? What things could be done to make you feel safer at school?

**BREAK**

**DRAWING THE IDEAL SCHOOL TECHNIQUE *(*INTERVIEWER DISCRETION AROUND WHICH CHILDREN THIS ACTIVITY IS DEVELOPENTALLY APPROPRIATE FOR)***

[For interviewer: Use flexibility with this approach, follow the student/child’s lead, note that some children may want to draw, others may prefer to write key words, and others may want to do both – do not prompt here; allow the child to decide what works best for them].

For this next activity, I will ask you to do some drawings, and I will be doing the writing, or you can draw and write if you like. You don’t need to include lots of details and it doesn’t matter if you make any mistakes.

***Part 1: Drawing the kind of school you would NOT like***

*The school*

Think about the kind of school you would not like to go to. This is not a real school. Make a quick drawing of this school in the middle of this paper. Tell me three things about this school. What kind of school is this?

*The classroom*

Think about the sort of classroom you would not like to be in, a place that was not comfortable. Make a quick drawing of this classroom in the school. Draw some of the things in this classroom.

*The students*

Think about some of the students at the school you would not like to go to. For example, if these students were an animal what might they be, a tiger or a cat; a wolf or a friendly dog? Make a quick drawing of some of these students. What are the students doing? Tell me three things about these students?

*The adults*

Think about some of the adults at the school you would not like to go to. Make a quick drawing of some of these adults. What are the adults doing or what have they done that was not comfortable for you? Tell me three things about these adults.

*Me*

Think about the kind of school you would not like to go to. Make a quick drawing of what you would be doing at this school. Tell me three things about the way you feel at this school.

***Part 2: Drawing the kind of school you would LIKE***

*The school*

Think about the kind of school you would like to go to. This is not a real school but an imaginary school, like YOUR kind of school if you could choose one. Make a quick drawing of this school in the middle of this paper. Tell me three things about this school. What kind of school is this?

*The classroom*

Think about the sort of classroom you would like to be in. Make a quick drawing of this classroom in the school. Think about where in the school in the classroom is located (e.g., near the library). Draw some of the things in this classroom.

*The students*

Think about some of the students at the school you would like to go to. Make a quick drawing of some of these students. What are the students doing? Tell me three things about these students.

*The adults*

Think about some of the adults at the school you would like to go to. Make a quick drawing of some of these adults. What are the adults doing (e.g., talking to the class, showing you how to do an activity, standing next to you)? Tell me three things about these adults.

*Me*

Think about the kind of school you would like to go to. Make a quick drawing of what you would be doing at this school. Tell me three things about the way you feel at this school.

**Next steps**

That’s all my questions. Thanks so much for speaking with me. Once we have finished seeing all the young people and speaking to parents and teachers, we will try put everything together and write a report. We will write a report for you too so that you know what everyone said about life at school. collate the data and try to pull it all together. At the end of the project, we will send you a report describing what the project was about, what we did, what we found and what it all means. In the meantime, if you have any further questions or would like to contact me for anything else, here are my contact details.

**End of interview**
