# Supplementary material for: “I Just Feel Like the Teacher Understood Me, and She Knew What I Needed”: School Experiences of Autistic Students from Diverse Backgrounds
Source: Autism Dev Lang Impair. 2025 Oct 15;10:23969415251377973. doi: 10.1177/23969415251377973 (PMC12535640; doi:10.1177/23969415251377973)
Supplement: sj-docx-2-dli-10.1177_23969415251377973 - Supplemental material for “I Just Feel Like the Teacher Understood Me, and She Knew What I Needed”: School Experiences of Autistic Students from Diverse Backgrounds [file sj-docx-2-dli-10.1177_23969415251377973.docx]

**DRAWING THE IDEAL SCHOOL TECHNIQUE: Instructions For Parents**

**Aim**: We would like your child to draw a picture of two schools:

1. A school they would **not like** to go to (example a)
2. A school they would **like** to go to (example b)

This is a very flexibility activity so your child may like to *draw* their schools, they might like to *write* key words about their schools, or they may like to do *both*.

**Materials**: pencils/pens and paper

**General tips:**

- Ensure your child knows there are no right or wrong ways of doing this activity. Any and all attempts should be encouraged.
- Encourage stick figures/sketches for children who may be more detail-orientated.
- Repeat instructions as often as necessary.
- Instructions below are a guide for what to say. Change language as required for your own child.
- Take breaks as needed.
- Praise your child’s effort throughout.
- If your child does not enjoy/want to write, you could write for your child. However, if you do write for them, ensure you write exactly what they say.

**PART A: Drawing the kind of school your child would not like to go to**

1. Ask your child to think about the kind of school they would **not like** to go to. Remind them that this is not a real school. You could say:

“*Make a quick drawing of this school in the middle of this paper. Tell me three things about this school. What kind of school is this?”*.

1. Encourage them to give you some details about the school, for example:

***The classroom:*** Ask them to think about the sort of classroom they would **not like** to be in, a place that was not comfortable. You could ask them to:

*“Make a quick drawing of this classroom in the school. Draw some of the things in this classroom if you would like.”*

***The students****:* Ask them to think about some of the students at the school they would **not like** to go to. For example, you could ask them:

*“If these students were an animal what might they be, a tiger or a cat; a wolf or a friendly dog? Make a quick drawing of some of these students. What are the students doing? Tell me three things about these students?”*

***The adults*:** Ask them to think about some of the adults at the school they would **not like** to go to. You could ask them to:

*“Make a quick drawing of some of these adults. What are the adults doing or what have they done that was not comfortable for you? Tell me three things about these adults.”*

***Themselves:*** Ask them to think about the kind of school they would **not like** to go to. You could ask them to:

*“Make a quick drawing of what you would be doing at this school. Tell me three things about the way you feel at this school.”*

Once your child has finished their drawing of a school they would **not like** to go do, encourage them to *“Draw the opposite, draw an ideal school, a school you would* ***like*** *to be at?”.*

**PART B: Drawing the kind of school your child would like to go to**

1. Ask your child to think about the kind of school they would **like** to go to. Remind them that this is not a real school. You could say:

“*Make a quick drawing of this school in the middle of this paper. Tell me three things about this school. What kind of school is this?”*.

1. Encourage them to give you some details about this second school, for example:

***The classroom:*** Ask them to think about the sort of classroom they would **like** to be in, a place that was comfortable. You could ask them to:

*“Make a quick drawing of this classroom in the school. Think about where in the school the classroom is located (e.g., near the library). Draw some of the things in this classroom.*

***The students****:* Ask them to think about some of the students at the school they would **like** to go to. For example, you could ask them:

*“Make a quick drawing of some of these students. What are the students doing? Tell me three things about these students.”*

***The adults*:** Ask them to think about some of the adults at the school they would **like** to go to. You could ask them to:

*“Make a quick drawing of some of these adults. What are the adults doing (e.g., talking to the class, showing you how to do an activity, standing next to you)? Tell me three things about these adults.”*

***Themselves:*** Ask them to think about the kind of school they would **like** to go to. You could ask them to:

*“Make a quick drawing of what you would be doing at this school. Tell me three things about the way you feel at this school.”*

**THIS IS THE END OF THE ACTIVITY.**

We would like a copy of your child’s drawings for this project. So, please either take photos or scan copies and email them to the email addresses below. Alternatively, we can provide you with a pre-paid envelope to send them back to us.

[EMAIL ADDRESSES]
